# Perspective: Advancing the science regarding temporomandibular disorders

**DOI:** 10.3389/fdmed.2024.1374883

**Published:** 2024-05-16

**Authors:** Richard Ohrbach, Alexandre F. DaSilva, Mildred C. Embree, John W. Kusiak

**Affiliations:** ^1^ Department of Oral Diagnostic Sciences, University at Buffalo School of Dental Medicine, Buffalo, NY, United States; ^2^ Headache and Orofacial Pain Effort (H.O.P.E.) Laboratory, Department of Biologic and Materials Sciences, University of Michigan School of Dentistry, Ann Arbor, MI, United States; ^3^ Cartilage Biology and Regenerative Medicine Laboratory, College of Dental Medicine, Columbia University Irving Medical Center, New York, NY, United States; ^4^ Santa Fe, NM, United States

**Keywords:** temporomandibular disorders, precision medicine, temporomandibular joint, research methods, science transfer

## Abstract

This Special Issue was initiated in response to the call for improved research by the National Academies of Sciences, Engineering, and Medicine (NASEM) (United States) Consensus Study Report on Temporomandibular Disorders (TMDs), a set of putatively localized musculoskeletal conditions. In this Special Issue, the importance of systems biology for TMDs emerges from each of three separate publications. The importance of systems biology to patients is anchored in two domains—laboratory research and clinical observation. The three publications fully speak to the underlying goals in the NASEM recommendations for initiatives: that research on TMDs needs to broaden, that integration between basic and clinical science needs to improve, and that while better evidence is needed, clinicians need to utilize the evidence that already exists. All three of these initiatives, taken together, would lead to better understanding of these complex diseases and to better care of patients with these diseases.

## 1 Introduction

The publication of *Temporomandibular Disorders: Priorities for Research and Care* (1) by NASEM in the United States stimulated, among many initiatives, the call for papers for this Special Issue on temporomandibular disorders (TMDs). In particular, this Special Issue was warranted in recognition of TMDs as complex diseases: traditional research approaches are no longer considered sufficient to advance our knowledge regarding these disorders and their treatment. We (the editors) were cognizant of innovative research studies being conducted on the masticatory system and on other systems relevant to improving our knowledge base regarding TMDs. Two themes for this issue were announced: application of systems biology to basic TMD research, and integration of basic and clinical research expertise. Invitations (more than 100) were mailed, using a variety of lists of known researchers and laboratories, five manuscripts were submitted, and three were accepted.

Two of the three accepted manuscripts meeting the objectives for this Special Issue involve basic science investigating osteoarthritis (OA) of the temporomandibular joint (TMJ) (2, 3). These two manuscripts nicely fulfilled the first theme. Indeed, those two papers represent the kind of science that the NASEM committee noted was urgently needed in order to change the trajectory of knowledge development regarding TMDs. Basic science of peripheral pain generators associated with musculoskeletal conditions, mechanisms underlying comorbidity, investigations of clinical phenotypes, improvements in diagnostic testing and nosology, role of behavior on musculoskeletal dysfunction, and rigorous clinical trials of existing or new therapies, among other important topics, are all missing from the final papers in this special issue. While these topics are clearly represented in the broader published pain research, we believe that too little of the research addresses the unique aspects of pain and mechanical problems associated with the masticatory system. For example, trigeminal-mediated nociception from the masticatory system appears to have unique significance for pain processing (4, 5), while the importance of mastication for survival appears to underly multidirectional possibilities in how an adaptive mechanism within the masseter muscles can be activated in order allow chewing and minimize pain (6). The absence of these listed topics in this Special Issue is a noteworthy gap and we believe that the gap points not only to the obvious research needs, but also to two other important aspects: the magnitude of the silo problem affecting both research and clinical care in this area of medicine and this area of the body, and the severe knowledge gap in implementing current pain science into new effective treatments.

Given the paucity of such science across the many topics essential for improved understanding of TMDs, it is perhaps not surprising that clinical beliefs and practices, as described by Greene and colleagues (7) in a Perspective that is the third paper in this issue, emerge to fill the knowledge gap. That such beliefs and practices do not accord with basic principles of disease epidemiology, known pathophysiology of TMDs, or clinical decision-making only serves to reinforce the views expressed in the NASEM report on TMDs: there is too little adequate science, and clinical practices do not adhere adequately to the science that does exist and is quickly evolving. Here, we will explore some immediate implications regarding current and future knowledge that is stimulated by the three science papers published in this Special Issue.

## 2 Current advances in basic science relevant to TMDs

### 2.1 Mechano-signaling is a local determinant for joint integrity

TMJ OA is a degradative joint disease resulting in tissue loss. Reed et al. (2) regard the pathogenesis of degenerative arthropathies, such as TMJ OA, as influenced by altered mechanical homeostasis. The extracellular matrix is speculated to initiate TMJ OA by transducing aberrant mechanical forces to TMJ fibrochondrocytes. However, the regulation of mechanical signals and the downstream events contributing to TMJ OA are poorly understood.

These studies demonstrate that the physical interaction between NG2/CSPG4, a transmembrane proteoglycan in TMJ fibrochondrocytes, and type VI collagen, a pericellular ECM protein, is critical for mechanical homeostasis and joint health. Using multiple *in vivo* and *in vitro* models Reed et al. demonstrate that NG2/CSPG deficiency and ERK 1/2 mechano-signaling is correlated with TMJ OA pathogenesis. For example, in a surgical TMJ OA mouse model, there is loss of NG2/CSPG4 and NG2/CSPG4-deficient mice develop TMJ OA. Using fibrochondrocytes, the application of static compression alters the ectodomain and increases the cytosolic NG2/CSPG4 intracelluar domain through clathrin-mediated endocytic pathways. RNA-seq and *in vitro* analyses reveal that NG2/CSPG4 deficient TMJ-derived chondrocytes have reduced mechanical activation of ERK 1/2 signaling, which contribute to enhanced expression of degradative proteases. Taken together, NG2/CSPG serves as a mechanistic link between the pericellular matrix and the intracellular signaling cascades that regulate aberrant fibrochondrocyte fates. These studies demonstrate the precise mechano-sensitive response pattern of fibrochondrocytes, highlighting the importance of controlled loading within a local mechanical environment for TMJ health.

The implication is that controlled reversal of TMJ degeneration may be possible. One hoped-for outcome of such treatment would be to restore a more healthy joint environment for proper signaling from joint afferents, for example as potential for improved proprioception (8) and its impact on restoring normal movement patterns, essential for normal mastication and speech. Knowledge gained from perhaps the most studied joint with OA—the knee—suggests that improvement in function following any medical treatment of knee OA is greatly enhanced by changing the forces on the joint through local exercise (9, 10) and that how such exercise is learned and nuances in its performance matter (11). We do not yet understand what corrective exercise for the masticatory system needs to be implemented in order to achieve true rehabilitative potential. The critical evaluation of clinical treatment methods for self-regulation and rehabilitation of masticatory function is only at the beginning (12, 13).

### 2.2 The determinants of anatomic structural integrity are not only local

Mackie et al. (3) used reliable measures of articular fossa and joint space to evaluate the effectiveness of machine-learning algorithms in diagnosing early to moderate stages of TMJ OA. They aimed to integrate biomarker, morphometry, and clinical data to improve imaging phenotypes. While the inclusion of fossa imaging biomarkers improved the performance of OA classifiers, these biomarkers alone may not diagnose early disease stages. The participants (*n* = 92) were examined in detail: DC/TMD examination; collection of serum and saliva biomarkers associated with nociception, inflammation, angiogenesis and bone resorption; and high-resolution cone beam computed tomography (h-CBCT) scans. The combined data were analyzed using two AI-based tools, TMJOAI (TMJ Osteoarthritis Artificial Intelligence) and TMJPI (TMJ Privileged Information), with the aim to create a more accurate, non-invasive diagnostic approach for TMJ OA, potentially leading to better patient care through early detection.

This study demonstrates notable advances in the use of technical tools for increasingly more granular assessment of bony structures associated with TMDs, but at the same time highlights the importance of clinical features—headaches, muscle soreness, and pain-free mouth opening—contributing to algorithmic classification. All three of these clinical features are overwhelmingly important constructs in the clinic, well-known to pain clinicians as sensitive markers of disease progress, and directly relevant to patient concerns. Identifying which individuals are likely to benefit from early conservative treatment approaches would represent a major conceptual advance for TMD treatments. Future longitudinal study assessments can use these baseline predictor values to build more sophisticated prediction models for estimating the risk of progression of TMJ osteoarthritis.

These additional important diagnostic features of headache, muscle soreness, and pain-free mouth opening all share the fundamental characteristic of pain and the influence of pain processing on how other non-pain findings (such as biomarkers related to the TMJ) manifest. Among multiple attributes—including domains of pain report, clinical tests, function, and beliefs—specific to the masticatory system, clinical measurements are influenced by the presence of other pain disorders, mood states, and general health (14). In other words, disease-specific attributes such as, for TMDs, the extent of pain-free jaw opening and its measurement do not exist separate from the generic or person level. Consequently, that Mackie and colleagues demonstrate the importance of clinical features for improving the algorithmic classification of TMJ OA is not surprising but, at the same time, is a critically important contribution to the principles necessary for an empirical taxonomy that encompasses the complexity of TMDs.

## 3 Discussion

### 3.1 Systems biology

Taken together, Mackie et al. and Reed et al. provide a pivotal set of findings supporting the current and increasingly used umbrella term for TMDs as a complex disease. A key feature of complex diseases is an intrinsic non-linearity between extent of a contributing mechanism and the output from that mechanism, where the non-linearity is a function of multi-level factors, reciprocal causation or dynamic feedback, phase transitions, or any combination (15, 16). Chronic pain disorders exhibit all of these mechanisms for non-linearity, and processes such as perceived stress contribute to the disorders in a complex manner that is typically idiosyncratic to the individual. Clearly, considering TMDs as complex diseases points to the limits of structure alone for classification. This is particularly true when the structure-based disease processes are intrinsically tied into other systems, for example, behavior and the effects of nociception (and pain) on the self (17).

The mounting evidence for the critical role of pain-related comorbidities, perhaps first expressed by Livingston (18) and subsequently expanded on by numerous studies (19–25) supporting risk for incidence, transition to chronic, and reciprocal causation within chronic pain states, highlights the tension between what is local for a given disorder, vs. what is generic or global to the person. That tension, in turn, reflects the clinical challenge of determining when peripheral findings related to a chronic pain disorder warrant direct forms of therapy, in contrast to a shift to central forms of therapy (26). Chronic pain is seldom accompanied by any evidence of local tissue problems sufficient to support inflammatory pain or nociceptive pain (27). This well-established observation is tempered by the potential presence of nociceptive drive that can continue to subserve sensitization and the full expression of pain processing (28). Moreover, such sensitization following tissue healing may readily represent the sufficient foci for inflammatory nociception—just not from the peripheral tissue (29). When should treatment for a local complaint not actually be local but rather focused on a mechanism related to pain with no evidential tissue damage? This question frames a current conundrum in how to balance attention to a complaint as perceived by the patient vs. clear directions emerging from good science. The findings from Mackie et al, in particular, point strongly towards an even more fundamental role of central mechanisms for classification of OA, vis-à-vis the clinical variables of headaches, muscle soreness, and pain-free mouth opening and their likely connections to the different parts of pain processing.

One further idea worth exploring, particularly in light of the importance of non-linear processes, is where, aside from pain processing, biology might meet “psychology” or, better, behavior in relation to TMJ pathophysiology and restoration of normal function. Any compromise to normal joint function—for example as mediated by early OA or problems in the disc-condylar relationship during mandibular movement—can be further exacerbated by the expectation of the individual that the joint should function normally. Sports injuries to a joint are perhaps the prototype for where expectation for recovery (and too-early resumption of athletic behaviors) often exceeds actual recovery and leads to impaired healing and delayed recovery (30–32). The well-established dysfunctional pattern of decreased behaviors described in the fear-avoidance model is countered by this behavioral pattern, no less dysfunctional, of increased behaviors based on unrealistic expectation of quick return of normal function in a joint. Expectation for normal functioning likely exerts its effects via top-down processing, affecting both behavioral activation as well as sensory processing (33). A better balance in the expectations of the functional capacity of an injured or compromised joint can affect, for example, outcomes after surgical interventions (34). New treatments for joint disease, as proposed by Reed et al., should not neglect the patient's psychological status. This is because it can have a significant impact on their behavior, which can either aid or hinder their rehabilitation. It is worth noting that clinicians who manage patients with musculoskeletal problems are now being taught to pay attention to both physical rehabilitation and mental attunement in the patient, especially in the TMD field (35).

### 3.2 Advancing the science

The manuscripts selected for our collection offer a perspective on the development of novel diagnostic and treatment science for clinical TMD context. Machine-learning algorithms can be optimized by incorporating both biological and pain-relevant data as early markers of disease; subsequent decision-making steps have the potential to improve prediction of TMJ OA progression and severity. And the responsiveness of TMJ OA to a novel therapeutic extracellular target, NG2/CSPG4, may ultimately be a function of better clinical phenotyping. While both studies show great potential to move the field forward, major questions persist on how diagnostic and therapeutic tools can offer superior outcomes compared to current clinical standards and provide substantial benefit in patient care.

The report by NASEM defined crucial steps needed to improve TMD care and research directions (1). One important consensus in the report is to match advances in science to clinical needs using a patient-centered perspective. However, for that, TMD science also has to better embrace patients' outcomes to provide solid evidence of safety and effectiveness in the long term (36). Some options include developing identified data elements and technological platforms that facilitate the standardization and analysis of large data that capture clinical, laboratory, and patient-generated data in an integrated ecosystem (37, 38), including the adoption of wearable and mobile technologies (39, 40). In addition, there is a need for the corresponding use of appropriate analysis methods for what can become multivariate within-person time-series data (41, 42) that will achieve better understanding of the chronic pain disorder as a process occurring within the patient's life (43, 44). Combining holistic approaches and precise data analysis with artificial intelligence from multiple teams will facilitate evidence-based science to reach more accurate diagnoses, successful treatment outcomes, and fewer mistakes in TMD practice. To accomplish that goal, however, the many implementation barriers to the actual collection of the necessary multi-dimensional data in the clinic will need to be addressed (45).

### 3.3 Future directions

Committed resources are needed to eliminate silos in basic and translational TMD research and bridge our discoveries with the clinic. Targeted governmental and foundational funding opportunities are approaches that could bolster these research efforts. One example might be a basic, interdisciplinary research initiative with a unique emphasis on interplay of temporomandibular pain and joint biomechanics. Another possibility would be a two-phase design and implementation program of a best practices approach to TMD treatments based on current basic, clinical, and behavioral discoveries in TMD-related research. We can envision an ongoing interprofessional educational seminar series in professional schools and continuing education programs providing up-to-date guidance on the changes in TMD clinical guidelines and standards of care as TMD research uncovers new knowledge about these disorders.

One final up-beat comment; we note that leadership at the National Institute of Dental and Craniofacial Research (NIDCR) at the National Institutes of Health (NIH) in the United States has developed an initiative is the TMD Collaborative for Improving Patient-Centered Translational Research (TMD IMPACT). This initiative aims to establish a national, interdisciplinary trans-NIH patient-centered collaboration to advance TMD basic and clinical research, research training, and translation to evidence-based treatments and improved clinical care. While this is a United States-based activity, TMD researchers in other countries note the same needs for their settings (46–48).

In summary, the statistics underlying these three published manuscripts highlight two important messages. One, there are many excellent laboratories involved with pain research but whether their research can help advance TMD knowledge remains unknown. And two, notwithstanding these excellent contributions, we believe that the research infrastructure for TMDs needs substantial support and improvement from universities and funding agencies in order to expand to the level needed for true advancement in this field.

## Data Availability

The original contributions presented in the study are included in the article/Supplementary Material, further inquiries can be directed to the corresponding author.

## References

[1] National Academies of Sciences Engineering and Medicine. Temporomandibular Disorders: Priorities for Research and Care. Washington, DC: The National Academies Press (2020). Available online at: https://www.nap.edu/catalog/25652/temporomandibular-disorders-priorities-for-research-and-care 32200600

[2] Reed DA Zhao Y Bagheri Varzaneh M Shin JS Rozynek J Miloro M NG2/CSPG4 Regulates cartilage degeneration during TMJ osteoarthritis. Front Dent Med. (2022) 3:1–18. 10.3389/fdmed.2022.1004942 PMC9850834 36685663

[3] Mackie T Al Turkestani N Bianchi J Li T Ruellas A Gurgel M Quantitative bone imaging biomarkers and joint space analysis of the articular Fossa in temporomandibular joint osteoarthritis using artificial intelligence models. Front Dent Med. (2022) 3:1–11. 10.3389/fdmed.2022.1007011 PMC9673279 36404987

[4] Uddin O Anderson M Smith J Masri R Keller A. Parabrachial complex processes dura inputs through a direct trigeminal ganglion-to-parabrachial connection. Neurobiol Pain. (2021) 9:100060. 10.1016/j.ynpai.2021.100060 33537510 PMC7840999

[5] Rodriguez E Sakurai K Xu J Chen Y Toda K Zhao S A craniofacial-specific monosynaptic circuit enables heightened affective pain. Nat Neurosci. (2017) 20(12):1734–43. 10.1038/s41593-017-0012-1 29184209 PMC5819335

[6] Minami I Akhter R Albersen I Burger C Whittle T Lobbezoo F Masseter motor unit recruitment is altered in experimental jaw muscle pain. J Dent Res. (2013) 92(2):143–8. 10.1177/0022034512470832 23242229

[7] Greene C Manfredini D Ohrbach R. Creating patients: how technology and measurement approaches are misused in diagnosis and convert healthy individuals into TMD patients. Front Dent Med. (2023) 4:1–7. 10.3389/fdmed.2023.1183327 PMC11797809 39916916

[8] Salamanna F Caravelli S Marchese L Carniato M Vocale E Gardini G Proprioception and mechanoreceptors in osteoarthritis: a systematic literature review. J Clin Med. (2023) 12(20):1–15. 10.3390/jcm12206623 PMC10607296 37892761

[9] Liu J Chen L Chen X Hu K Tu Y Lin M Modulatory effects of different exercise modalities on the functional connectivity of the periaqueductal grey and ventral tegmental area in patients with knee osteoarthritis: a randomised multimodal magnetic resonance imaging study. Br J Anaesth. (2019) 123(4):506–18. 10.1016/j.bja.2019.06.017 31395306

[10] Serrano-García B Forriol-Campos F Zuil-Escobar JC. Active neurodynamics at home in patients with knee osteoarthritis: a feasibility study. J Clin Med. (2023) 12(20):1–15. 10.3390/jcm12206635 PMC10607651 37892772

[11] Hunt MA Takacs J Hart K Massong E Fuchko K Biegler J. Comparison of mirror, raw video, and real-time visual biofeedback for training toe-out gait in individuals with knee osteoarthritis. Arch Phys Med Rehab. (2014) 95(10):1912–7. 10.1016/j.apmr.2014.05.016 24910929

[12] Story WP Durham J Al-Baghdadi M Steele J Araujo-Soares V. Self-management in temporomandibular disorders: a systematic review of behavioural components. J Oral Rehabil. (2016) 43(10):759–70. 10.1111/joor.12422 27487973

[13] Durham J Al-Baghdadi M Baad-Hansen L Breckons M Goulet J-P Lobbezoo F Self-management programmes in temporomandibular disorders: results from an international Delphi process. J Oral Rehabil. (2016) 43(12):929–36. 10.1111/joor.12448 27727477

[14] Sharma S Slade GD Fillingim RB Greenspan JD Rathnayaka N Ohrbach R. Attributes germane to temporomandibular disorders and their associations with five chronic overlapping pain conditions. J Oral Facial Pain Headache. (2020) 34(0):s57–72e. 10.11607/ofph.2582 32975541 PMC10073965

[15] Young G Chapman CR. Pain, affect, nonlinear dynamical systems, and chronic pain: bringing order to disorder. In: Young G Nicholson K Kane AW, editors. Causality of Psychological Injury: Presenting Evidence in Court. New York: Springer (2010). p. 197–241.

[16] Galea S Riddle M Kaplan GA. Causal thinking and complex system approaches in epidemiology. Int J Epidemiol. (2009) 39(1):97–106. 10.1093/ije/dyp296 19820105 PMC2912489

[17] Chapman CR Tuckett RP Song CW. Pain and stress in a systems perspective: reciprocal neural, endocrine, and immune interactions. J Pain. (2008) 9(2):122–45. 10.1016/j.jpain.2007.09.006 18088561 PMC2278005

[18] Livingston WK. Pain and Suffering. Seattle: IASP Press (1998).

[19] Visscher CM Schouten MJ Ligthart L van Houtem CM de Jongh A Boomsma DI. Shared genetics of temporomandibular disorder pain and neck pain: results of a twin study. J Oral Facial Pain Headache. (2018) 32:107–12. 10.11607/ofph.2016 29509827

[20] Favarato MH Germani ACCG Martins M. Glimpsing the raging seas that stop swans: a qualitative look at living with multimorbidity and pain in patients from a tertiary care service. J Multimorb Comorb. (2021) 11:2633556521999509. 10.1177/2633556521999509 33796473 PMC7968021

[21] Kleykamp BA Ferguson MC McNicol E Bixho I Arnold LM Edwards RR The prevalence of psychiatric and chronic pain comorbidities in fibromyalgia: an ACTTION systematic review. Semin Arthritis Rheu. (2021) 51(1):166–74. 10.1016/j.semarthrit.2020.10.006 33383293

[22] Teruel A Romero-Reyes M. Interplay of oral, mandibular, and facial disorders and migraine. Curr Pain Headache Rep. (2022) 26(7):517–23. 10.1007/s11916-022-01054-6 35567662

[23] Ohrbach R Fillingim RB Mulkey F Gonzalez Y Gordon S Gremillion H Clinical findings and pain symptoms as potential risk factors for chronic TMD: descriptive data and empirically identified domains from the OPPERA case-control study. J Pain. (2011) 12(11, Supplement 3):T27–45. 10.1016/j.jpain.2011.09.001 22074750 PMC3443556

[24] Sanders AE Slade GD Bair E Fillingim RB Knott C Dubner R General health status and incidence of first-onset temporomandibular disorder: the OPPERA prospective cohort study. J Pain. (2013) 14(12, supplement 2):T51–62. 10.1016/j.jpain.2013.06.001 24275223 PMC3855662

[25] Kleykamp BA Ferguson MC McNicol E Bixho I Arnold LM Edwards RR The prevalence of comorbid chronic pain conditions among patients with temporomandibular disorders: a systematic review. J Am Dent Assoc. (2021) 153(3):241–50. 10.1016/j.adaj.2021.08.008 34952681

[26] Kohns DJ Scott R Castellanos J Scribner D Hodges R Clauw DJ. The impact of nociplastic pain features on the response to physical therapy in patients with primary myofascial pain. J Back Musculoskelet. (2022) 35(5):1143–51. 10.3233/BMR-210244 35213348

[27] Gerhardt A Eich W Janke S Leisner S Treede RD Tesarz J. Chronic widespread back pain is distinct from chronic local back pain: evidence from quantitative sensory testing, pain drawings, and psychometrics. Clin J Pain. (2016) 32(7):568–79. 10.1097/AJP.0000000000000300 26379077

[28] Harper DE Schrepf A Clauw DJ. Pain mechanisms and centralized pain in temporomandibular disorders. J Dent Res. (2016) 95(10):1102–8. 10.1177/0022034516657070 27422858 PMC5004242

[29] Hawkins JL Durham PL. Prolonged jaw opening promotes nociception and enhanced cytokine expression. J Oral Facial Pain Headache. (2016) 30:34–41. 10.11607/ofph.1557 26817031 PMC5894825

[30] Arvinen-Barrow M Clement D Hamson-Utley JJ Kamphoff C Zakrajsek R Lee SM Athletes’ expectations about sport-injury rehabilitation: a cross-cultural study. J Sport Rehabil. (2016) 25(4):338–47. 10.1123/jsr.2015-0018 27632833

[31] Carroll LJ Lis A Weiser S Torti J. How well do you expect to recover, and what does recovery mean, anyway? Qualitative study of expectations after a musculoskeletal injury. Phys Ther. (2016) 96(6):797–807. 10.2522/ptj.20150229 26586855

[32] Podlog L Heil J Schulte S. Psychosocial factors in sports injury rehabilitation and return to play. Phys Med Rehabil Clin N Am. (2014) 25(4):915–30. 10.1016/j.pmr.2014.06.011 25442166

[33] Gilbert CD Sigman M. Brain states: top-down influences in sensory processing. Neuron. (2007) 54(5):677–96. 10.1016/j.neuron.2007.05.019 17553419

[34] Taylor CEV Murray CM Stanton TR. Patient perspectives of pain and function after knee replacement: a systematic review and meta-synthesis of qualitative studies. Pain Rep. (2022) 7(3):e1006. 10.1097/PR9.0000000000001006 35558092 PMC9088230

[35] Bahns C Scheffler B Kopkow C. Guideline-adherent physiotherapy for patients with hip and knee osteoarthritis in Germany: protocol for an implementation research project using the theoretical domains framework and the behavior change wheel. JMIR Res Protoc. (2023) 12:e47834. 10.2196/47834 37971802 PMC10690534

[36] Reid K Greene C. Diagnosis and treatment of temporomandibular disorders: an ethical analysis of current practices. J Oral Rehabil. (2013) 40(7):546–61. 10.1111/joor.12067 23691977

[37] Gressler LE Cowley T Velezis M Aryal S Clare D Kusiak JW Building the foundation for a modern patient-partnered infrastructure to study temporomandibular disorders. Front Dig Health. (2023) 5:1–19. 10.3389/fdgth.2023.1132446 PMC10226081 37255961

[38] Elstad E Bocell FD Owens TC Logan D Melluso E Viscione C Focus groups to inform the development of a patient-reported outcome measure (PROM) for temporomandibular joint disorders (TMDs). Patient. (2023) 16(3):265–76. 10.1007/s40271-023-00618-x 36840915 PMC9961303

[39] DaSilva AF Robinson MA Shi W McCauley LK. The forefront of dentistry-promising tech-innovations and new treatments. JDR Clin Trans Res. (2022) 7(1_suppl):16s–24s. 10.1177/23800844221116850 36121134 PMC9793430

[40] Kaciroti N DosSantos MF Moura B Bellile EL Nascimento TD Maslowski E Sensory-discriminative three-dimensional body pain mobile app measures versus traditional pain measurement with a visual analog scale: validation study. JMIR Mhealth Uhealth. (2020) 8(8):e17754. 10.2196/17754 32124732 PMC7468641

[41] In: Stone AA Turkkan JS Bachrach CA Jobe JB Kurzman HS Cain VS, editors. The Science of Self-Report: Implications for Research and Practice. Mahwah, NJ: Lawrence Erlbaum (2000) p. 1–393.

[42] Kaplan SEF Ohrbach R. Self-report of waking-state oral parafunctional behaviors in the natural environment. J Oral Facial Pain Headache. (2016) 30:107–19. 10.11607/ofph.1592 27128474

[43] Bäckryd E. Chronic pain and time—a theoretical analysis. J Pain Res. (2023) 16:4329–35. 10.2147/JPR.S435830 38145034 PMC10741732

[44] Tabor A Constant A. Lifeworlds in pain: a principled method for investigation and intervention. Neurosci Conscious. (2023) 2023(2):1–8. 10.1093/nc/niad021 PMC10499064 37711314

[45] Sharma S Breckons M Bronnimann-Lambelet B Chung JW List T Lobbezoo F Challenges in the clinical implementation of a biopsychosocial model for assessment and management of orofacial pain. J Oral Rehabil. (2019) 47(1):87–100. 10.1111/joor.12871 31398261

[46] Durham J Greene C Ohrbach R. A commentary on temporomandibular disorders: priorities for research and care—bridging from the US to the UK. Brit Dent J. (2022) 233(3):232–3. 10.1038/s41415-022-4501-6 35962105 PMC9372950

[47] Peck C Greene C Ohrbach R. Lessons for Australia—commentary on “temporomandibular disorders: priorities for research and care”. Aust Dent J. (2023) 68(1):3–6. 10.1111/adj.12940 36302509

[48] Oyarzo J Jusakos M Greene C Ohrbach R. Observaciones sobre trastornos temporomandibulares:prioridades de investigación y atención: ¿cómo avanzará Chile? Medwave. (2023) 23(1):1–5. 10.5867/medwave.2023.01.2648 36883888

